# Effects of Commercial Copper Nanoparticles on Enzymatic Activity and Nutrient Bioavailability in Agricultural Soil

**DOI:** 10.3390/nano16211394

**Published:** 2026-10-30

**Authors:** Jonathan Suazo-Hernández, Paula Maldonado, Erick Zagal, María de Los Ángeles Sepúlveda-Parada, Antonieta Ruiz, Cristian Urdiales, Collin G. Joseph, Lizethly Cáceres-Jensen, Patricia Poblete-Grant

**Affiliations:** 1 Núcleo de Investigación en Biotecnología y Agroinnovación (NIBA), Escuela de Agronomía, Facultad de Medicina Veterinaria y Agronomía, Universidad de Las Américas, Sede Concepción, Concepción 4030000, Chile; 2 VIS-IR Spectroscopy Laboratory and Sustainable Soil Management, Department of Soils and Natural Resources, Faculty of Agronomy, University of Concepción, Chillán 3812120, Chile; pmaldonado2019@udec.cl (P.M.); ezagal@udec.cl (E.Z.); mariadelosasepu@udec.cl (M.d.L.Á.S.-P.); 3 Doctoral Program in Agronomy Sciences, Faculty of Agronomy, Universidad de Concepción, Chillán 3812120, Chile; 4 Department of Chemical Sciences and Natural Resources, Universidad de La Frontera, Avenida Francisco Salazar, P.O. Box 54-D, Temuco 01145, Chile; maria.ruiz@ufrontera.cl; 5 Sede Vallenar, Universidad de Atacama, Av. Costanera #105, Vallenar 1612178, Chile; cristian.urdiales@uda.cl; 6 Sonophotochemistry Research Group, Faculty of Science and Technology, Universiti Malaysia Sabah, Kota Kinabalu 88400, Sabah, Malaysia; collin@ums.edu.my; 7 Industrial Chemistry Programme, Faculty of Science and Technology, Universiti Malaysia Sabah, Kota Kinabalu 88400, Sabah, Malaysia; 8 Laboratorio de Fisicoquímica & Analítica, PachemLab, Departamento de Química, Facultad de Ciencias Básicas, Universidad Metropolitana de Ciencias de la Educación, Santiago 7760197, Chile; lyzethly.caceres@umce.cl; 9 Centro de Nanociencia y Nanotecnología, CEDENNA, Santiago, Chile; 10 Departamento de Acuicultura y Recursos Agroalimentarios, Campus Osorno-Chuyaca, Universidad de los Lagos, Osorno 5311157, Chile; patricia.pobletegrant@ulagos.cl

**Keywords:** nano-pollution, soil properties, nano-agrochemicals, sustainable development goals

## Abstract

Excessive release of engineered nanoparticles (NPs) into the environment can affect the biological and chemical properties of soils. This study evaluated the effects of two commercial metallic copper nanoparticles (Cu-NPs 25 nm; CuNPs/25, and Cu-NPs 40–60 nm; CuNPs/40–60) on enzymatic activity and nutrient bioavailability in an agricultural soil. An incubation experiment was conducted using Ultisol soil treated with 500 mg kg^−1^ CuNPs/25 or CuNPs/40–60, simulating a high-exposure scenario, in the presence and absence of 200 mg kg^−1^ P (K_2_HPO_4_). Acid phosphatase, alkaline phosphatase, and β-glucosidase enzymatic activities along with P bioavailability were measured at 0.14, 1, 3, 5, and 8 weeks. The results showed that in most instances, 500 mg kg^−1^ CuNPs/25 or CuNPs/40–60 significantly decreased the activities of acid phosphatase, alkaline phosphatase, and β-glucosidase. The P bioavailability in soil was lower in the presence of CuNPs/25 than Cu-NPs/40–60. In addition, the Cu^2+^ concentrations in the water-extractable fraction were higher than in the CaCl_2_ fraction, and the increase in water-extractable Cu^2+^ concentrations in the soil solution was correlated with a decrease in enzyme activities. Eight weeks of exposure to NPs did not cause any marked changes in organic matter content or electrical conductivity. However, the Cu^2+^-(DTPA) bioavailability in soil treated with 500 mg kg^−1^ CuNPs/25 and CuNPs/40–60 for 8 weeks increased over 90 times, regardless of the presence or absence of P. However, these findings should be interpreted in the context of a high-exposure scenario for CuNPs and should not be directly extrapolated to environmentally realistic exposure levels.

## 1. Introduction

In recent decades, nanotechnology has experienced a massive expansion, reflected in the increased production and use of engineering nanoparticles (NPs). Estimates suggest that the NP market will increase from USD 8.78 million in 2025 to USD 115.41 billion by 2034 [1]. This growth is due to the greater surface-to-volume ratio and surface reactivity of nanoscale materials compared to their bulk counterparts [2], which improves their mechanical, magnetic, and optical properties [3].

Currently, NPs play a vital role in the development and economic growth of various industrial sectors, such as construction, electronics, medicine, cosmetics, agriculture, and environmental protection [4]. In particular, nano-agrochemical formulations of pesticides and fertilizers, especially those formulated with metallic copper nanoparticles (CuNPs), have seen significant application in modern agriculture [5]. For example, nanopesticide use is predicted to grow from 1.40 million tons in 2024 to 2.17 million tons by 2034 [6]. This economic increase is due to the potent antibacterial properties of CuNPs, which are sustained for extended periods due to the gradual release of Cu^2+^ cations [5,7]. This intrinsic property of CuNPs allows for reduced costs, increased material lifespans, improved agricultural productivity and crop nutrition, and decreased environmental pollution [8], even under drought stress conditions [9]. For all these reasons, it has been suggested that CuNPs could be very helpful in achieving the 17 sustainable development goals (SDGs) set by the United Nations to address climate change, resource scarcity, and feeding the growing global population [10].

On the other hand, Cu^2+^ is a micronutrient for plants when its concentration in plant tissues is between 5 and 20 mg kg^−1^ [11]. However, when the concentration of Cu^2+^ exceeds 20 mg kg^−1^, it can affect plant growth and development and therefore impact crop production and ultimately food security [11]. Therefore, massive input of CuNPs into the environment can increase Cu^2+^ concentrations in soil and negatively impact plant productivity and, consequently, agroecosystems [12]. Thus, intensive application of CuNPs in the form of nanopesticides or nanofertilizers can result in a vast amount of CuNPs remaining unreacted and accumulating on the soil surface, where they can interact with biotic and abiotic systems [13]. Furthermore, CuNPs tend to have longer residence times in soils than in other environmental matrices [2]. Consequently, soil matrices have been identified as the main sink for NPs [14]. Therefore, to ensure food safety and human health, it is crucial to understand the environmental impact of CuNPs in soils before their widespread agricultural application.

Current data on the interactions of NPs with abiotic and biotic soil components are varied [15]. Some research indicates that NPs can have beneficial and negative effects on the biological, physical, and chemical properties of soils, depending primarily on the type, size, surface chemistry, and concentration of the NPs, as well as the physical and chemical properties of the soil [16,17,18,19,20].

Soil enzyme activity plays a key role in biological processes, such as the degradation of pollutants, nutrient cycling, the breakdown of organic matter (OM), and crop production. Enzymes allow for efficient nutrient transfer and are fundamental to maintaining soil health. However, it has been demonstrated that urease and cellulase [21], β-1,4-N-acetylglucosaminidase [22], and dehydrogenase [23] activities are inhibited in the presence of CuNPs. A decrease in enzymatic activity can severely impair overall soil health, limiting the essential functions of soil microbiota enzymes [24]. Therefore, monitoring soil enzyme activities can provide information on soil health as they are sensitive to changes caused by anthropogenic factors, including the use of NPs [25,26].

Regarding soil chemical properties, metallic CuNPs can modify pH, cation exchange capacity, nutrient bioavailability, and increase electrical conductivity [17,27]. In addition, CuNPs can release Cu^2+^ ions, increasing their bioavailable concentration in the soil solution over time [17,21,28]. For example, in a volcanic soil treated with 10,000 mg kg^−1^ CuNPs, the extractable Cu^2+^-(DTPA) fraction was much higher (720 mg kg^−1^) than that in the soil without NPs (2.08 mg kg^−1^) [27]. An increase in the bioavailable Cu^2+^ concentration in the soil solution can lead to increased toxicity to soil microorganisms, and altered speciation and adsorption/aggregation of nutrients such as P, thereby altering their equilibria and, consequently, their dynamics [17]. In agricultural soils, this phenomenon could force farmers to apply higher concentrations of P fertilizers [29].

Although there are many studies on the impact of CuNPs on enzymatic processes and nutrient bioavailability, and the solubilization/dissolution of CuNPs to Cu^2+^ in soils, there is a significant gap regarding published research on Chilean soils. In addition, there are no studies on different particle sizes of CuNPs, resulting in a lack of data on their impact on soil biological and chemical properties. We hypothesized that CuNPs 25 nm and CuNPs 40–60 nm have different effects on soil enzymatic activity and P bioavailability of soil due to their differences in surface characteristics. The objective of this study was to evaluate the effects of two commercial CuNPs with different particle size on enzymatic activity and P bioavailability in a Chilean Ultisol. Moreover, the changes in pH and concentration of Cu^2+^ released from CuNPs were also measured.

## 2. Materials and Methods

### 2.1. Acquisition and Characterization of Nanoparticles

Commercial CuNPs (25 nm and 40–60 nm) in fine powder form were acquired from Sigma-Aldrich (CAS number 7440–50–8; St. Louis, MO, USA).

The CuNPs/25 and CuNPs/40–60 were characterized by X–Ray Diffraction (XRD) at the wavelength of CuKα radiation (1.5418 Å) in the 2θ region of 20–80° using a Bruker D8 VENTURE diffractometer (Bruker AXS, Karlsruhe, Germany). The specific surface area (SSA) of CuNPs/25 and CuNPs/40–60 was determined using the Brunauer–Emmett–Teller (BET) model and the pore diameter and pore volume by applying the Barrett–Joyner–Halenda (BJH) theory following conducting N_2_ adsorption–desorption experiments at liquid nitrogen on a Flex Micromeritics analyze (Norcross, GA, USA). For each ENP, about 1.0 g of dry powder was outgassed for about 15 h at 150 C (7 × 10^−6^ atm) before performing the measurement [30].

### 2.2. Collection and Characterization Soil Sample

Ultisol soil, according to the USDA soil taxonomy for agricultural use, was collected from the south-central region of Chile at 37°42′54.5″ S 72°37′09.8″ W [31]. Before soil sampling, the surface mulch layer and visible plant residues were carefully removed. Soil samples were collected from the A horizon (0–20 cm depth) following a zig-zag sampling design and combined into a single sample [32]. The soil was kept at room temperature until use.

The initial pH of the soil was determined using a 1:2.5 soil:double-distilled water solution. Electrical conductivity (EC) in the soil was measured using a 1:5 soil:water solution. The OM content in the soil was measured using 20 mL of potassium dichromate (K_2_Cr_2_O_7_), while sulfuric acid (H_2_SO_4_) was used as the oxidizing agent [33]. NH_4_^+^-N and NO_3_^−^-N were extracted from the soil using 2 mol L^−1^ potassium chloride (KCl) and measured using the MgO method [34]. Available nitrogen (N) in the soil was calculated as the sum of the NH_4_^+^-N and NO_3_^−^-N contents. Bioavailable phosphorus (P_olsen_) was extracted with 0.5 mol L^−1^ sodium bicarbonate (NaHCO_3_) at pH 8.50 and measured using the ammonium molybdate ((NH_4_)_6_Mo_7_O_24_) method at 880 nm [35] with a Rayleigh UV-2601 spectrophotometer (BRAIC Co., Ltd., Beijing, China). The exchangeable K in the soil was extracted with a 1.0 mol L^−1^ ammonium acetate (NH_4_CH_3_CO_2_) solution at pH 7.0 and subsequently measured using atomic absorption spectroscopy (AAS; iCE™ 3300, Thermo Scientific, Waltham, MA, USA) [33]. Copper (Cu) bioavailability in the soil was determined using diethylenetriaminepentaacetic acid (DTPA) extraction [36] and measured by AAS. The apparent and bulk densities of the soil samples were determined using the cylinder method [37]. The sand, silt, and clay contents were determined according to the method used by Sandoval et al. [38]. The effects of CuNPs/25 and CuNPs/40–60 on the EC, OM content, and Cu^2+^-(DTPA) bioavailability in the soil were also analyzed at the end of the incubation experiment. These properties were measured as described above.

### 2.3. Incubation Studies

To simulate the impact of CuNPs/25 or CuNPs/40–60 on soil health, the Ultisol soil was exposed to CuNPs/25 or CuNPs/40–60 in the presence or absence of P (K_2_HPO_4_) for 8 weeks. The concentrations used were 0 and 500 mg kg^−1^ for CuNPs/25 or CuNPs/40–60 and 0 and 200 mg kg^−1^ for P. Therefore, six treatments were applied: Soil, Soil + P, Soil + CuNPs/25, Soil + CuNPs/40–60, Soil + P + CuNPs/25, and Soil + P + CuNPs/40–60. A previous study realized by Adhikari et al. [39] that investigated NP toxicity thresholds and assessed soil resilience under conditions of high NP accumulation suggests that 500 mg kg^−1^ CuNPs represents a high-exposure or worst-case scenario. The 200 mg kg^−1^ P concentration was chosen because it falls within the range of phosphate concentrations applied to agricultural soils in southern Chile [40]. Therefore, the selected treatments aimed to simulate real P bioavailability conditions and allowed us to evaluate whether its presence modulates the effects of high NP concentrations on soil properties.

In this study, the soil was hydrated to field capacity and then left at 20 C for two weeks to activate soil microorganisms. Afterwards, the temperature was increased to 22 C, marking the start of the incubation. The effect of NPs on the activity of three enzymes was evaluated at different time weeks following exposure to the various treatments. In addition, P bioavailability and Cu^2+^ released from the NPs were analyzed at the different time points using different extraction agents.

### 2.4. Enzymatic Activities

Enzymatic activities in the Soil, Soil + P, Soil + CuNPs/25, Soil + CuNPs/40–60, Soil + P + CuNPs/25, and Soil + P + CuNPs/40–60 systems were evaluated at 0.14, 1, 3, 5, and 8 weeks. The activities of acid phosphatase and alkaline phosphatase, markers of the phosphorus cycle, were measured using the protocol established by Tabatabai and Bremner [41]. In this procedure, 1.0 g of soil was treated with a solution of p-nitrophenyl phosphate disodium (pNPP) as the substrate, and then incubated at 37 C for 1 h. Subsequently the p-nitrophenol (PNP) released from pNPP hydrolysis was measured at 400 nm using a Rayleigh UV-2601 spectrophotometer (BRAIC Co., Ltd., Beijing, China). The activity of β-glucosidase, an enzyme associated with the carbon cycle, was measured using the protocol established by Tabatabai [42]. The phosphatase activities and β-glucosidase activities were expressed as µmol PNP g^−1^ soil h^−1^.

### 2.5. Extractable Cu^2+^ and Final pH

The Cu^2+^ concentration in the Soil, Soil + P, Soil + CuNPs/25, Soil + CuNPs/40–60, Soil + P + CuNPs/25, and Soil + P + CuNPs/40–60 systems was determined at 0.14, 1, 3, 5, and 8 weeks using water and CaCl_2_ as the extraction agents [43]. For water extraction, 1.0 g of soil was mixed with 10 mL of distilled water and then shaken in a reciprocating shaker at 180 rpm for 2 h. For CaCl_2_ extraction, 1.0 g of soil was mixed with 10 mL of 0.01 mol L^−1^ CaCl_2_ and then shaken at 180 rpm for 180 min. Later on, the samples were centrifuged at 6000 rpm for 10 min. The pH of the supernatant (water and CaCl_2_ extracts) was measured using an Thermo Fisher Scientific 710A Orion pH/ISE meter (Waltham, MA, USA) with a combination glass electrode.

The supernatants were added to a falcon tubes and 100 µL of concentrated HNO_3_ was added to acidify the samples to ensure that the Cu^2+^ remained in solution rather than forming a precipitate [44]. Subsequently, the supernatant was filtered using 0.45 µm pore-size filter membranes. Finally, the concentration of Cu^2+^ in the solution was quantified using AAS (iCE TM 3300, Thermo Scientific, Waltham, MA, USA).

### 2.6. Phosphorus Bioavailability

To evaluate the effect of CuNPs/25 or CuNPs/40–60 on P bioavailability, the P concentration was quantified in the Soil, Soil + P, Soil + CuNPs/25, Soil + CuNPs/40–60, Soil + P + CuNPs/25, and Soil + P + CuNPs/40–60 systems at 0.14, 1, 3, 5, and 8 weeks. P was extracted from the soil samples using a 0.5 mol L^−1^ NaHCO_3_ solution at pH 8.50, and the samples were then shaken for 30 min at 180 rpm [33]. The solutions were filtered, and the P in the extract was measured at 880 nm [35] using a Rayleigh UV-2601 spectrophotometer (BRAIC Co., Ltd., Beijing, China).

### 2.7. Statistical Analysis

The treatments were applied to independent soil samples, and the reported replicates correspond to biological replicates. Each replicate consisted of an independent experimental unit that was prepared, treated, and incubated separately but under identical conditions. Enzymatic and nutrient analyses were performed in technical triplicate, and the mean value was used in the subsequent statistical analysis [45].

Prior to statistical analysis, the assumptions of normality and homogeneity of variances were evaluated using the Shapiro–Wilk and Levene’s tests, respectively. Statistical analyses were performed separately for each incubation time point to evaluate the effect of treatment on the measured variables. For each incubation time point, differences between treatments were assessed using one-way analysis of variance (ANOVA), considering treatment as the fixed factor. When significant treatment effects were detected (*p* ≤ 0.05), mean separation was performed using Tukey’s honestly significant difference (HSD) post hoc test.

All statistical analyses were conducted in R (version 4.6.0) using the packages tidyverse for data manipulation, car for testing homogeneity of variances, rstatix for statistical analysis, agricolae for Tukey HSD comparisons and compact letter display, and ggplot2 for graphical representation. The results are presented as the mean ± standard error (SE), and different letters indicate statistically significant differences between treatments at the same incubation time point (*p* ≤ 0.05).

In addition, variables were standardized using z-score transformation and the relationships between alkaline and acid phosphatase activities and Cu concentrations extracted with water (H_2_O) or CaCl_2_ were evaluated using Pearson’s correlation coefficient (r). Simple linear regression was used to visualize the relationships, with the fitted regression line and its 95% confidence interval displayed. Statistical significance was assessed using the corresponding *p*-values, with significance set at *p* ≤ 0.05.

## 3. Results and Discussion

### 3.1. NPs Characterization

**Figure 1:**
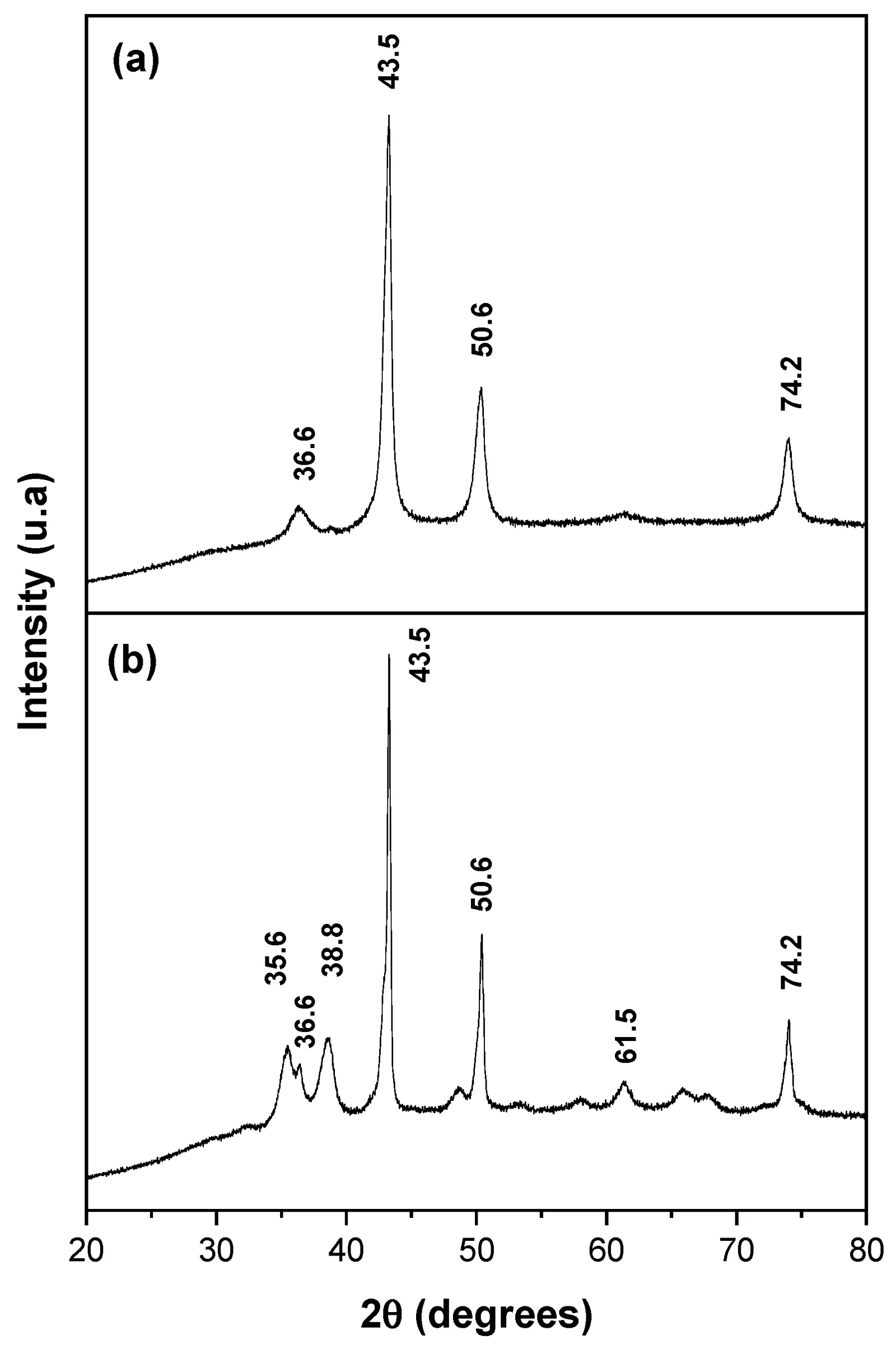
XRD analysis of (**a**) CuNPs/25 and (**b**) CuNPs/40–60.

Figure 1 shows the XRD analyses of CuNPs/25 and CuNPs/40–60. In Figure 1a, CuNPs/25 exhibited peaks at 2θ = 43.5°, 50.6°, and 74.2°, which are associated with the elemental Cu structure, while the peak at 2θ = 36.6° is associated with the Cu_2_O structure [29,46]. Meanwhile, for CuNPs/40–60 (Figure 1b), the four peaks observed for CuNPs/25 were also detected, and, in addition, three new peaks were observed at 2θ = 35.6°, 38.8° and 61.5°, which are characteristic of the CuO structure [28,47]. Therefore, CuNPs/40–60 exhibited a higher degree of oxidation than CuNPs/25.

The CuNPs/40–60 had higher values of SSA, pore volume, and pore diameter than CuNPs/25 (Table 1), which suggests that CuNPs/25 are undergoing an aggregation process due to electrostatic and van der Waals forces [48,49].

### 3.2. Soil Properties

Table 2 shows the physicochemical properties of the Ultisol soil used. The percentages of sand, silt, and clay in the soil were 39.0%, 33.4%, and 27.6%, respectively, which are characteristic of a clay loam soil [50]. The soil had a pH of 6.3, which is slightly acidic, and a moderate OM content (2.1%), suggesting a moderate nutrient retention capacity and biological activity [51]. The EC was 0.10 dS m^−1^, indicating a very low concentration of soluble salts in the soil, classifying it as a non-saline soil.

The available N content was 11.3 mg kg^−1^ and was mainly present as NH_4_^+^ (10.5 mg kg^−1^), suggesting that the mineral soil composition favors NH_4_^+^ retention over nitrification. The bioavailable P (P_Olsen_) content in the soil was 5.2 mg kg^−1^, which could promote plant growth [51]. The exchangeable K^+^ content was 0.23 cmol+ kg^−1^, indicating that there is a low amount of available K^+^ [51]. The Cu^2+^(DTPA) content was 2.02 mg kg^−1^, suggesting that the soil contained a high level of this element [52]. This is relevant because at low concentrations, Cu^2+^ is vital for plant growth and development. In fact, Cu^2+^ can serve as a structural cofactor for enzymes involved in photosynthesis and respiration. However, an excess of Cu^2+^ in soil can cause oxidative stress in plants [53].

### 3.3. Effect of Nanoparticle Size on Enzymatic Activity

**Figure 2:**
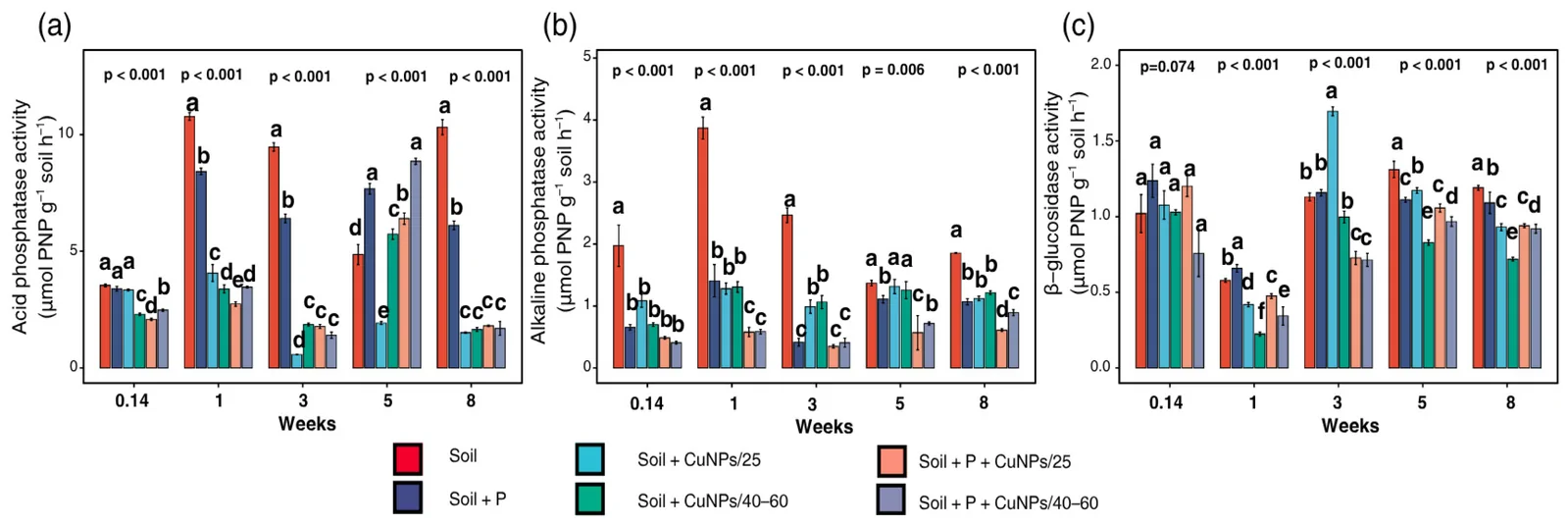
(**a**) Acid phosphatase, (**b**) alkaline phosphatase, and (**c**) β-glucosidase activities in agricultural soil after different incubation times with the following treatments: Soil, Soil + P, Soil + CuNPs/25, Soil + CuNPs/40–60, Soil + P + CuNPs/25, and Soil + P + CuNPs/40–60. Bars represent means ± standard error (SE). Different lowercase letters indicate significant differences among treatments within each sampling time according to Tukey’s multiple comparison test (*p* ≤ 0.05). Treatments sharing at least one letter are not significantly different. The *p*-values displayed above each sampling time indicate the overall treatment effect.

Soil microbial enzymes play a fundamental role in soil biological processes, such as the degradation and mineralization of organic compounds, and nutrient cycling, including P cycling [54]. Furthermore, enzymes respond to environmental disturbances, making them potential indicators of microbial community health [23]. The activities of acid phosphatase, alkaline phosphatase, and β-glucosidase showed marked variation across CuNPs treatments and incubation times (Figure 2).

In particular, acid phosphatase activity plays an important role in the hydrolysis of organic phosphorus to orthophosphate ions in acidic soils [55]. The highest acid phosphatase activity was observed with the Soil treatment, except for 5 weeks, reaching values of 3.53, 10.84, 9.54, and 9.27 μmol PNP g^−1^ h^−1^ at 0.14, 1, 3, and 8 weeks, respectively (Figure 2a). At 0.14 weeks for all treatments decreased acid phosphatase activity compared with the control Soil treatment, with significant reductions observed with the Soil + CuNPs/40–60, Soil + P + CuNPs/25, and Soil + P + CuNPs/40–60 treatments (*p* ≤ 0.05). At 1, 3 and 8 weeks, the decrease in acid phosphatase activity was significant for all treatments compared with the Soil control. This behavior may reflect a reduced requirement for enzymatic mineralization in the presence of an external P source [55,56]. In addition, 500 mg kg^−1^ CuNPs/25 or CuNPs/40–60, alone and in combination with P, decreased acid phosphatase activity, suggesting that the presence of NPs and/or the Cu^2+^ cations released from the NPs may contribute to enzymatic activity inhibition. One possible explication is the antimicrobial activity of Cu^2+^, which could reduce microbial activity and consequently decrease enzyme production or release into the soil [57,58]. Alternatively, Cu^2+^ may interact directly with extracellular enzymes, potentially reducing their activity [21]. However, these mechanisms were not directly evaluated in the present study and should therefore be considered plausible explanations rather than demonstrated causal pathways.

On the other hand, the control Soil, Soil + P, Soil + CuNPs/25, Soil + CuNPs/40–60, Soil + P, CuNPs/25 and Soil + P + CuNPs/40–60 treatments showed lower alkaline phosphatase activity (≤3.33 μmol PNP g^−1^ h^−1^) compared to acid phosphatase activity over time (Figure 2b). At 0.14 weeks, all treatments showed significantly lower alkaline phosphatase activity than the Soil control (*p* ≤ 0.05), with the Soil + P + CuNPs/40–60 treatment showing the lowest activity value. Similarly, at 1, 3, 5 and 8 weeks, the Soil + P + CuNPs/25 and Soil + P + CuNPs/40–60 treatments showed the lowest alkaline phosphatase activities, and at 8 weeks the reductions for Soil + P + CuNPs/25 and Soil + P + CuNPs/40–60 was approximately 67% and 52% compared with the control Soil treatment. These results indicate that NP size, high NP concentrations and the presence of P affect soil alkaline phosphatase activity and are consistent with those values obtained for silt–clay–loam soils from Saricalar in the presence of CuONPs [59].

The β-glucosidase enzyme plays an important role in the degradation of cellulose, and thus, in the global carbon cycle and turnover of soil OM [60]. In the experiment, β-glucosidase activity varied with treatment and incubation time (Figure 2c). In general, significant variations were observed after 1 week (*p* ≤ 0.05). The β-glucosidase activity of the control Soil treatment at 1 week was 0.58 μmol PNP g^−1^ h^−1^. Meanwhile, treatment with NPs, especially CuNPs/40–60, had a significant decrease (*p* ≤ 0.05), with Soil + CuNPs/40–60 treatment having a more persistent inhibitory effect on β-glucosidase activity compared to CuNPs/25, reaching a value of 0.22 μmol PNP g^−1^ h^−1^. The combination of 500 mg kg^−1^ CuNPs and P tended to attenuate the decrease in β-glucosidase activity over longer incubation periods (>3 weeks), although this effect varied according to NP size. In particular, the notable decrease in acid and alkaline phosphatase activities over the time and under the high concentration of CuNPs treatment (500 mg kg^−1^) may reflect effects on microbial activity and/or direct interactions between Cu^2+^ species and soil enzyme [21]. However, changes in microbial community structure were not evaluated and therefore cannot be confirmed. Overall, the observed enzymatic responses suggest that P input and exposure to high CuNP concentrations may influence soil biological activity.

### 3.4. Effect of Nanoparticle Size on Extractable Cu^2+^

**Figure 3:**
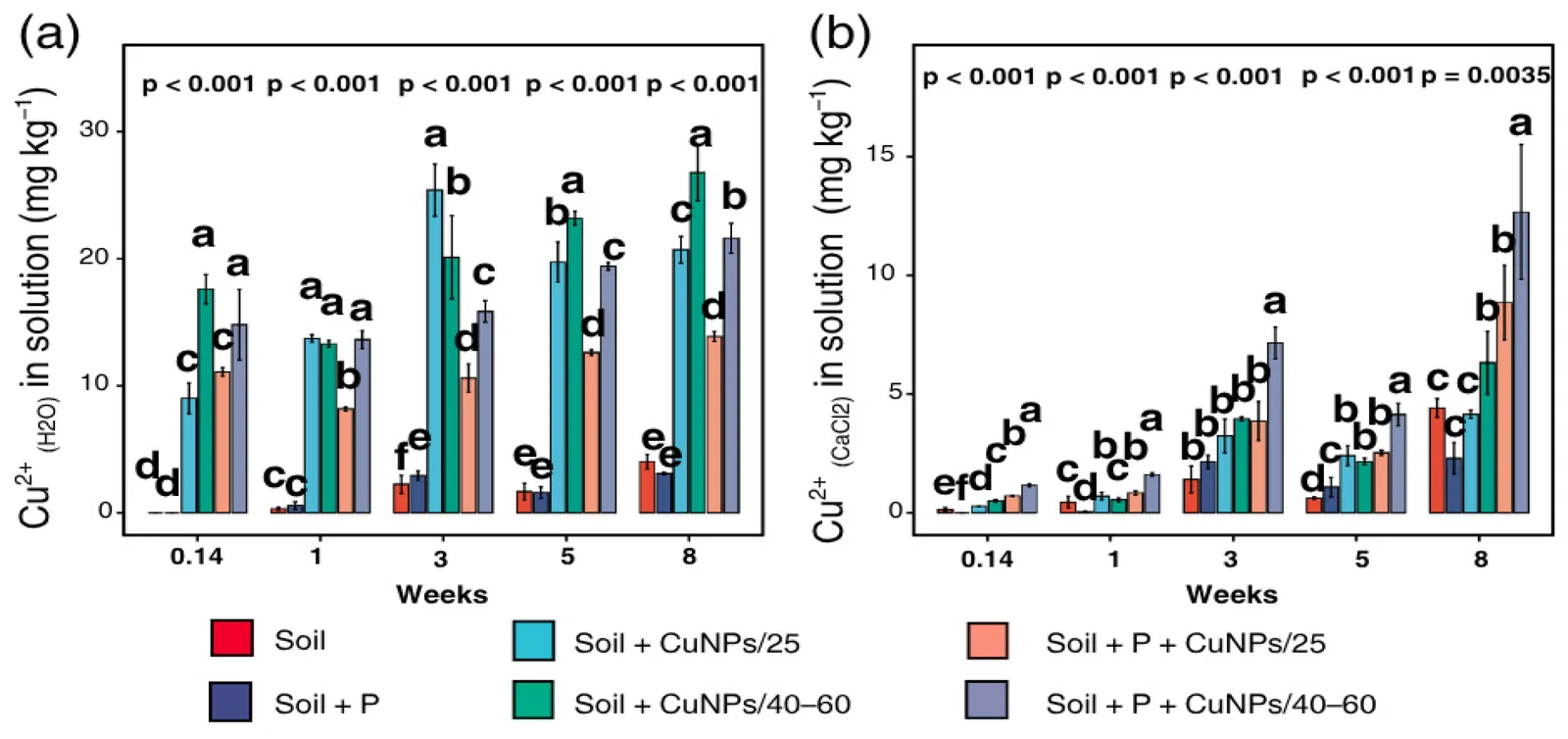
Available Cu^2+^ in agricultural soil extracted using (**a**) water and (**b**) CaCl_2_ after different incubation times with the following treatments: Soil, Soil + P, Soil + CuNPs/25, Soil + CuNPs/40–60, Soil + P + CuNPs/25, and Soil + P + CuNPs/40–60. Bars represent means ± standard error (SE). Different lowercase letters indicate significant differences among treatments within each sampling time according to Tukey’s multiple comparison test (*p* ≤ 0.05). Treatments sharing at least one letter are not significantly different. The *p*-values displayed above each sampling time indicate the overall treatment effect.

Agricultural soil is a heterogeneous matrix, which gives it a high affinity for Cu^2+^. This makes it difficult for dispersants to bind to and extract Cu^2+^ from the soil and also significantly increases the variability in the results obtained using different extractants [61] (Figure 3).

The water-extractable and CaCl_2_-extractable fractions contain “labile Cu^2+^” and they are indicators of the mobility and bioavailability of Cu^2+^ for soil biota and plant growth [62].

Figure 3 shows that the water- and CaCl_2_-extractable Cu^2+^ concentrations increased over time [22]. In addition, the Cu^2+^ concentration in the water-extractable fraction was higher than in the 0.01 mol L^−1^ CaCl_2_-extractable fraction for all treatments. This suggests that water can mobilize free Cu^2+^ and Cu^2+^ associated with labile compounds, while CaCl_2_ primarily extracts the exchangeable fraction, which is more limited [63]. However, it is also possible that the CaCl_2_ solution inhibited the release of Cu^2+^ from CuNPs by reducing electrostatic repulsion, thereby promoting NP aggregation and reducing NP dissolution [64,65]. In general, the water-extractable Cu^2+^ concentration showed a progressive increase over time with all the treatments (Figure 3a), except for Soil + P + CuNPs/40–60 treatment at 1 week and Soil + CuNPs/25 treatment at 3 weeks. This trend could be related to a slower and sustained dissolution of the CuNPs, which increased the concentration of Cu^2+^ in solution over time. In addition, CuNPs/40–60 had a larger SSA (8.71 m^2^ g^−1^), and consequently, more reaction sites. This property may have caused CuNPs/40–60 to dissolve to a greater extent and may have favored higher Cu^2+^ release, supporting the results obtained at 0.14, 5 and 8 weeks and being in line with Xu et al. [44]. In addition, those values were lower for Soil + P + CuNPs/25 and Soil + P + CuNPs/40–60 than Soil + CuNPs/25 and Soil + CuNPs/40–60 treatments, which suggests that under that conditions CuNPs were more stable. The explanation could be attributed to the i) Cu^2+^ released from NPs is more strongly adsorbed onto soil solid phases in the presence of P than without P and ii) P could serve as a Lewis base that donates electron pairs, binding Cu atoms and forming highly stable complexes [66]. In volcanic soils, it has been demonstrated that the Cu^2+^ released from CuNPs can interact with phosphate (PO_4_^3−^), forming a precipitate consisting of Cu_3_(PO_4_)_2_ [29]. In summary, there are many possible explanations for the variation in extractable Cu^2+^ concentration over time.

The trend for Cu^2+^ concentration in the CaCl_2_-extractable fraction over time was 8 > 3 > 5 > 1 > 0.14 weeks (Figure 3b). However, the Cu^2+^ concentrations were several times lower than those in the water-extractable fraction. Therefore, the low amount of Cu^2+^ obtained in the CaCl_2_-extractable fraction may provide some indication of the extractable amount of Cu^2+^ released from the NPs and adsorbed onto soil surfaces, representing the fraction that more easily bioavailable to soil microorganisms and plants [57,62].

On the other hand, using water- and CaCl_2_ as extraction agents, the time required for the complete dissolution of NPs added to soil in high amounts could range from months to years [67], which is associated with NPs concentration and soil properties [65]. In this sense, Sekine et al. [68] demonstrated that in the first few days, soil pH directly influences the solubilization of NPs. Similarly, at later time points, soil properties (e.g., pH, OM content, and clay content) influence NP transformations.

### 3.5. Effect of Nanoparticle Size on pH

**Figure 4:**
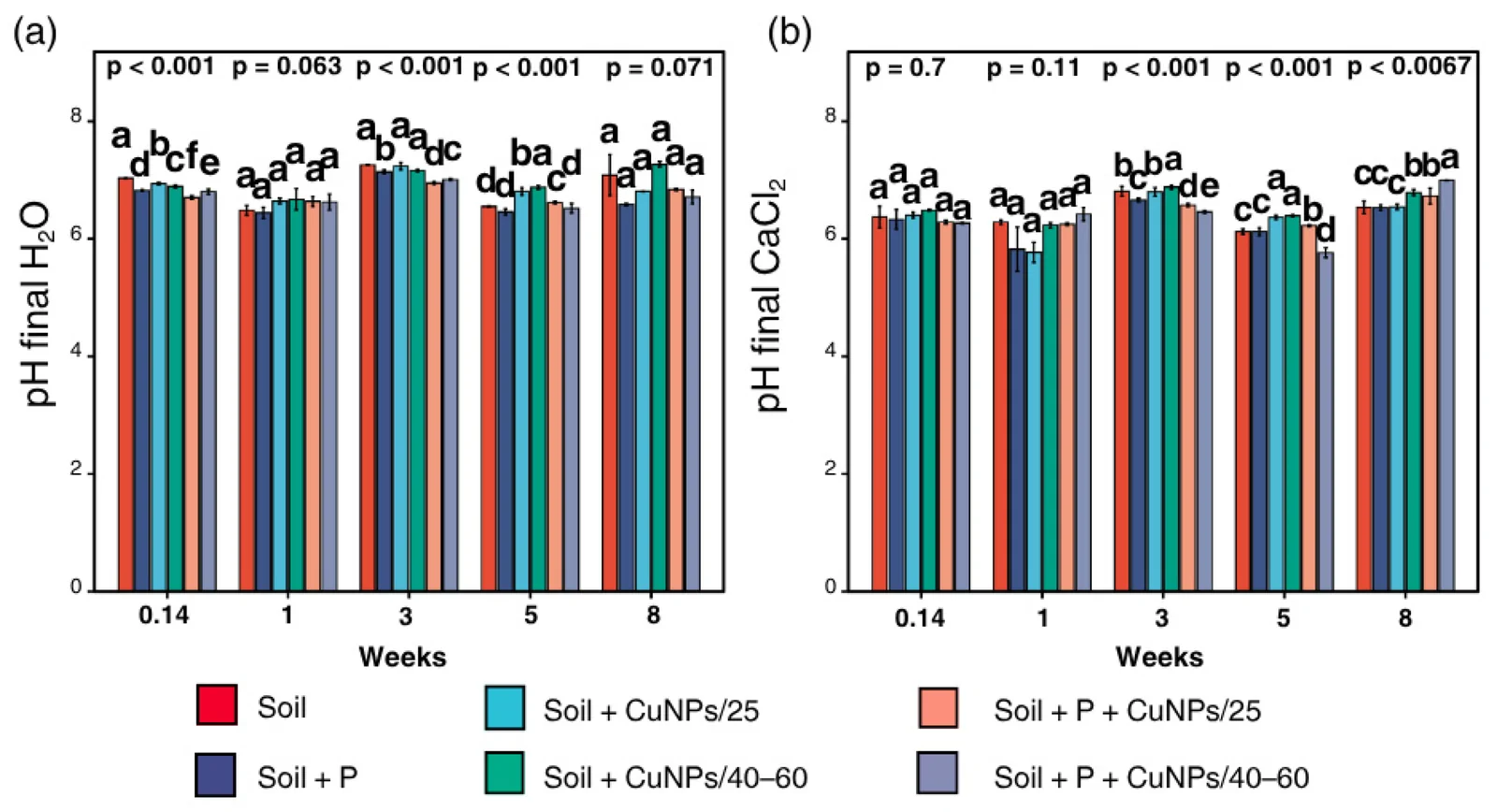
pH_Final_ of (**a**) water- and (**b**) CaCl_2_-extracted fractions from agricultural soil after different incubation times with the following treatments: Soil, Soil + P, Soil + CuNPs/25, Soil + CuNPs/40–60, Soil + P + CuNPs/25, and Soil + P + CuNPs/40–60. Bars represent means ± standard error (SE). Different lowercase letters indicate significant differences among treatments within each sampling time according to Tukey’s multiple comparison test (*p* ≤ 0.05). Treatments sharing at least one letter are not significantly different. The *p*-values displayed above each sampling time indicate the overall treatment effect.

Soil pH showed minimal variation between treatments and incubation times in the water- and CaCl_2_-extracted fractions (Figure 4). In general, the pH of the water-extracted fraction in the control Soil treatment was significantly different at 0.14, 3, and 5 weeks (Figure 4a). Meanwhile, the pH of the CaCl_2_-extracted fraction was significantly different at 3, 5, and 8 weeks (Figure 4b). At 0.14 weeks, the pH of the water-extracted fraction for the different treatments ranged between 6.7 and 7.0, and at 8 weeks, it ranged between 6.6 and 7.3.

The control Soil treatment showed a slight tendency toward lower pH values during the first week, indicating a modest increase in acidity that persisted throughout the incubation period (Figure 4a). In general, Soil treatment with CuNPs (Soil + CuNPs/40–60 and Soil + CuNPs/25) resulted in a slight decrease in the pH of the water-extracted fraction, especially after 0.14 weeks, which could be attributed to salt hydrolysis processes or H^+^ release during the interaction of CuNPs/Cu^2+^ with the soil matrix [69]. Similar pH variations were observed by Kachel et al. [70] for CuNPs in a cultivated soil from eastern Poland, Lublin Province. This suggests that in short times, the applied NP concentration did not drastically alter the pH, thereby maintaining favorable conditions for nutrient bioavailability and biological activity [71]. However, at 5 weeks, the pH of the water-extracted fraction significantly increased in the presence of 500 mg kg^−1^ CuNPs/25 or CuNPs/40–60 (Soil + CuNPs/40–60 and Soil + CuNPs/25) compared with the control. This may be related to complexation of H^+^ at the oxide surface, which could also contribute to an increase in pH if the zero charge point of the metal oxide is higher than the soil pH.

### 3.6. Effect of Nanoparticle Size on Phosphorus Bioavailability

**Figure 5:**
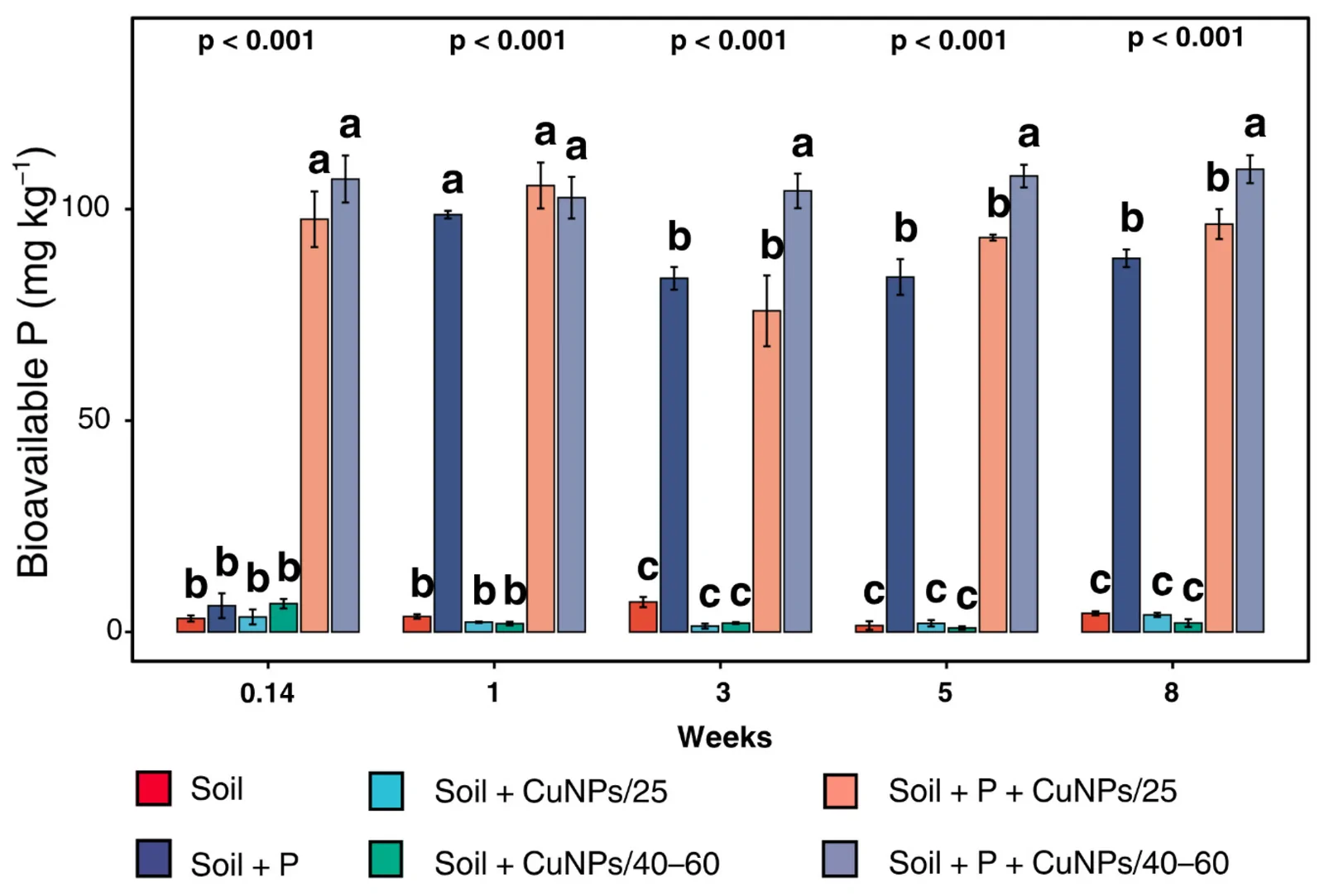
Bioavailable P in agricultural soil after different incubation times with the following treatments: Soil, Soil + P, Soil + CuNPs/25, Soil + CuNPs/40–60, Soil + P + CuNPs/25, and Soil + P + CuNPs/40–60. Bars represent means ± standard error (SE). Different lowercase letters indicate significant differences among treatments within each sampling time according to Tukey’s multiple comparison test (*p* ≤ 0.05). Treatments sharing at least one letter are not significantly different. The *p*-values displayed above each sampling time indicate the overall treatment effect.

The temporal dynamics of P bioavailability revealed significant differences between treatments and incubation times (*p* ≤ 0.05), as shown by the distinct letter groupings in Figure 5. Between 0.14 and 8 weeks, the control Soil treatment had a bioavailable P concentration ≤ 7.1 mg kg^−1^, with no significant variation at ≤1 week, but after 3 weeks, the changes were significant. In particular, at 0.14 weeks, treatment with 200 mg kg^−1^ P (Soil + P) significantly increased the bioavailable P concentration, reaching values of 6, 99, 83, 84, and 88 mg kg^−1^ at 0.14, 1, 3, 5, and 8 weeks, respectively. Treatment with CuNPs (Soil + CuNPs/25 and Soil + CuNPs/40–60) showed bioavailable P concentrations ranging from 1 to 12 mg kg^−1^ over time, with the highest levels observed at 8 weeks. The combination of P and CuNPs (Soil + P + CuNPs/25 and Soil + P + CuNPs/40–60) maintained relatively high concentrations of bioavailable P throughout the incubation period, although slight differences between NP sizes and sampling times were observed. The highest bioavailable P concentrations were observed after 1 week of the treatments with P, particularly Soil + P + CuNPs/40–60. This data could be associate with the higher release of Cu^2+^ from CuNPs/40–60 (Figure 3), which may modify P speciation and its interaction with the soil solid phase [72], to a greater extent than CuNPs/25, potentially contributing to a higher bioavailability, as determined by NaHCO_3_ extraction (Figure 5).

The results also showed that increasing the incubation time significantly decreased P bioavailability in the Soil + P and Soil + P + CuNPs/25 treatments (*p* ≤ 0.05) compared with the same treatment at 1 week. In this sense, Cu^2+^ have a high affinity for P, forming insoluble complexes, which can lead to the immobilization of Cu^2+^ in soils with available P [73]. A similar trend was observed in a saline soil [74] and volcanic soils [29]. In agricultural volcanic soils, this may mean that farmers who use CuNP agrochemicals need to consider adding higher concentrations of phosphate fertilizers to meet plant demands. Overall, the grouping of treatments highlights the distinct interactions between Cu^2+^ species and soil P, which influence the bioavailability of P.

### 3.7. Effect of Nanoparticle Size on Chemical Properties

The OM content, EC, and Cu^2+^ bioavailability were measured at the end of the incubation time (Table 3).

After 8 weeks, the OM content was relatively similar between the different treatments, suggesting that the addition of CuNPs/25 or CuNPs/40–60 and P did not alter this soil parameter. Similar results were obtained by Shi et al. [75] in paddy soils treated with CuONPs for 90 days. The EC (dS m^−1^) of the Ultisol soil after 8 weeks was the same as the initial value (Table 3). The Soil + CuNPs/25 and Soil + CuNPs/40–60 treatments showed similar EC values to the control Soil treatment. In contrast, Soil + P and Soil + CuNPs/40–60, especially the Soil + P + CuNPs/25 treatment, showed slightly higher EC values compared with the Soil treatment. Since EC is a measure of the salt and ion contents in the solution [76], these data revealed that P and NPs caused the increase in EC. On the other hand, Cu^2+^-(DTPA) bioavailability increased by about 90.5 and 99.5 times with CuNPs/25 or CuNPs/40–60, respectively, compared with the Soil treatment (Table 3). This effect was more pronounced with CuNPs/40–60, which was associated with a higher oxidation grade (Figure 1). These results are particularly relevant because concentrations above 150 mg kg^−1^ of Cu^2+^-(DTPA) in soil represent a potential risk to plant growth and soil health [77].

Meanwhile, the combination of NPs and P increased Cu^2+^-(DTPA) bioavailability by 93.6 (Soil + P + CuNPs/25) and 113.9 (Soil + P + CuNPs/40–60) times compared with the Soil control treatment. This interaction suggests that the presence of P may influence Cu^2+^ bioavailability and mobility in soils treated with NPs.

**Figure 6:**
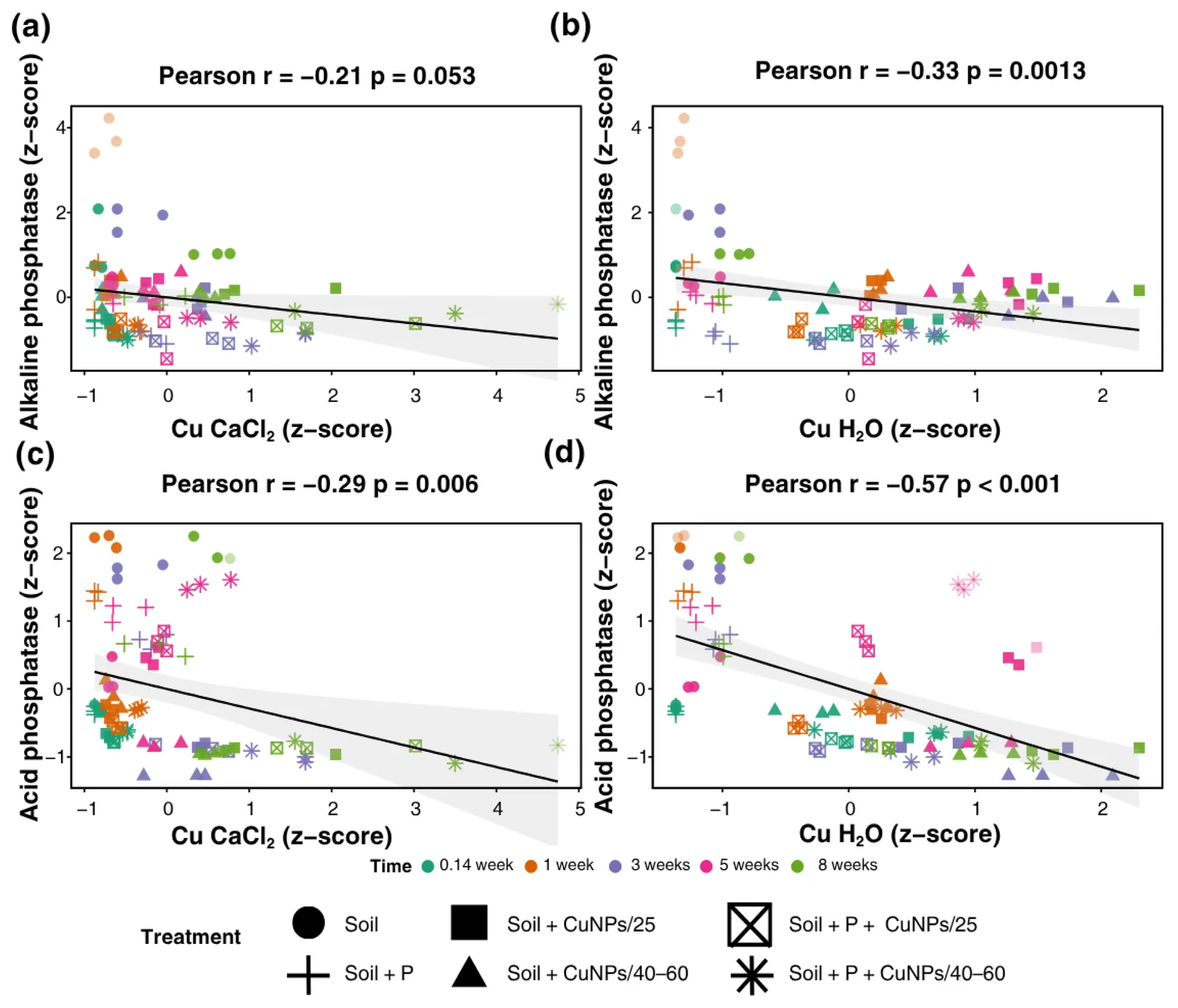
Relationship between soil Cu extractability and phosphatase activities across phosphorus (P) fertilization and copper nanoparticle (CuNPs) treatments during eight weeks of incubation. (**a**) Alkaline phosphatase activity versus CaCl_2_-extractable Cu, (**b**) alkaline phosphatase activity versus water-extractable Cu, (**c**) acid phosphatase activity versus CaCl_2_-extractable Cu, and (**d**) acid phosphatase activity versus water-extractable Cu. All variables were standardized using z-scores. Colors indicate incubation times (0.14, 1, 3, 5, and 8 weeks), while symbols distinguish the six experimental treatments: Soil (circle), Soil + P (plus sign), Soil + CuNPs/25 (square), Soil + CuNPs/40–60 (triangle), Soil + P + CuNPs/25 (crossed square), and Soil + P + CuNPs/40–60 (asterisk). Solid black lines represent fitted linear regression models, and shaded areas indicate 95% confidence intervals generated using the default settings in R. Pearson’s correlation coefficients (r) and their associated *p*-values are reported for each relationship.

Figure 6 shows negative correlations between extractable Cu (using CaCl_2_ and water) and acid and alkaline phosphatase activities. In particular, for alkaline phosphatase, the relationships were weak to moderate, with the strongest association observed with water-extractable Cu^2+^ (r = −0.33; *p* = 0.0013). For acid phosphatase, the trend was more pronounced, especially with water-extractable Cu^2+^ (r = −0.57; *p* ≤ 0.001). Therefore, the water-extractable fraction could have a stronger inhibitory effect on enzymatic activity, and, in addition, may explain the lower enzymatic activities observed for CuNPs/40–60 than for CuNPs/25 during most incubation weeks (Figure 2), as discussed above.

Similarly, Adhikari et al. [39] also found that acid phosphatase inhibition was negatively correlated with water-extractable Cu^2+^. However, CuNPs/25 or CuNPs/40–60 can undergo dissolution, aggregation, agglomeration, and oxidation processes, and interact with soil OM and mineral surfaces over time. Consequently, the decreased enzymatic activities could stem from the properties of the NPs and/or the transformed species generated during soil incubation, rather than solely from the higher concentration of Cu^2+^ in the soil solution. Therefore, it is important to emphasize that the results obtained in Ultisol must be interpreted with caution.

## 4. Social Implications and Relevance for Agricultural Sustainability

The increasing use of NPs, particularly CuNPs, in agricultural practices can pose significant social challenges related to the sustainability of food production, soil health, and environmental safety. Since agricultural soils form the basis of food production, any alteration in their biological and chemical properties, such as changes in enzyme activity, nutrient bioavailability, or pH, can have a direct impact on crop productivity and, consequently, on the economic and social stability of human beings [78]. In this regard, it is essential to understand the impact of commercial CuNPs with different particle sizes on the biological and chemical properties to know the risks and benefits associated with their massive application in agricultural soils, especially in contexts of intensified production.

From a social perspective, the inadequate management of CuNPs contained in nano-agrochemicals could lead to negative effects, such as phytotoxic, ecotoxicological, and food chain transfer effects. These risks are particularly relevant for farmers, who have limited access to technical information [79]. The eventual degradation of soil and health quality by Cu-based nano-agrochemicals can compromise the economic sustainability of producers and increase their dependence on external inputs. Overall, despite the relatively high CuNP concentrations used, this study is scientifically relevant, providing essential information for developing sustainable and resilient agricultural systems that are aligned with current and future social needs.

## 5. Conclusions

This study showed that, in most cases, the application of 500 mg kg^−1^ of commercial CuNPs/25 or CuNPs/40–60 in Ultisol soil was associated with a significant decrease in acid phosphatase, alkaline phosphatase, and β-glucosidase enzymatic activities, accompanied by an increase in water-extractable Cu^2+^ concentrations. These findings supported an association between Cu^2+^ availability and enzymatic responses; however, the specific mechanisms for enzyme inhibition could not be established based on the present results. Therefore, the potential environmental risks associated with NP application (e.g., as a component of nano-agrochemicals, especially nano-fungicides) require future research, including potential effects on other microbiological properties (e.g., microbial community diversity), plant productivity, and abiotic conditions (e.g., salinity, drought, and low- and high-temperature stress) due to exposure to different NP concentrations.

## 6. Declaration of Generative AI in Scientific Writing

During the preparation of this work, the main author used M365 copilot (GPT-5) to review the final manuscript. The main author takes full responsibility for the content of the published article.

## Figures and Tables

**Table 1:** Specific surface area, pore volume, and pore diameter of nanopaticles.

Property	CuNPs/25	CuNPs/40–60
Specific surface area (mg g^−1^)	7.42	8.71
Pore volume (cm^3^ g^−1^)	0.0402	0.0487
Pore diameter (nm)	31.1	35.1

**Table 2:** Physicochemical properties of the soil used.

Parameter	Value
pH	6.3
Electrical conductivity (dS m^−1^)	0.10
Organic matter (%)	2.1
NO_3_^−^-N (mg kg^−1^)	0.8
NH_4_^+^-N (mg kg^−1^)	10.5
Available N (mg kg^−1^)	14.3
Bioavailable P (mg kg^−1^)	5.2
Exchangeable K^+^ (cmol+ kg^−1^)	0.23
Cu-(DTPA) (mg kg^−1^)	2.02
Apparent density (g cc^−1^)	1.54
Real density (g cc^−1^)	2.52
Sand (%)	39.0
Silt (%)	33.4
Clay (%)	27.6

**Table 3:** Organic matter (OM) content, electrical conductivity (EC), and bioavailable Cu^2+^ concentration in soil at the end of the incubation experiment.

Soil Property	Soil	Soil + P	Soil + CuNPs/25	Soil + CuNPs/40–60	Soil + P + CuNPs/25	Soil + P + CuNPs/40–60
OM (%)	2.10 ± 0.07	2.10 ± 0.00	2.05 ± 0.07	2.10 ± 0.00	2.00 ± 0.00	2.00 ± 0.00
EC (dS m^−1^)	0.10 ± 0.00	0.20 ± 0.00	0.10 ± 0.00	0.10 ± 0.00	0.30 ± 0.00	0.20 ± 0.00
Cu^2+^-(DTPA) (mg kg^−1^)	2.02 ± 0.14	2.20 ± 0.00	183 ± 1.41	201 ± 1.41	189 ± 1.41	230 ± 0.00

## Data Availability

The raw data generated in this study will be made available by the authors on request.
